# Supplementary figures and images for: Expression and Function of C1orf132 Long-Noncoding RNA in Breast Cancer Cell Lines and Tissues
Source: Int J Mol Sci. 2021 Jun 23;22(13):6768. doi: 10.3390/ijms22136768 (PMC8268529; doi:10.3390/ijms22136768)

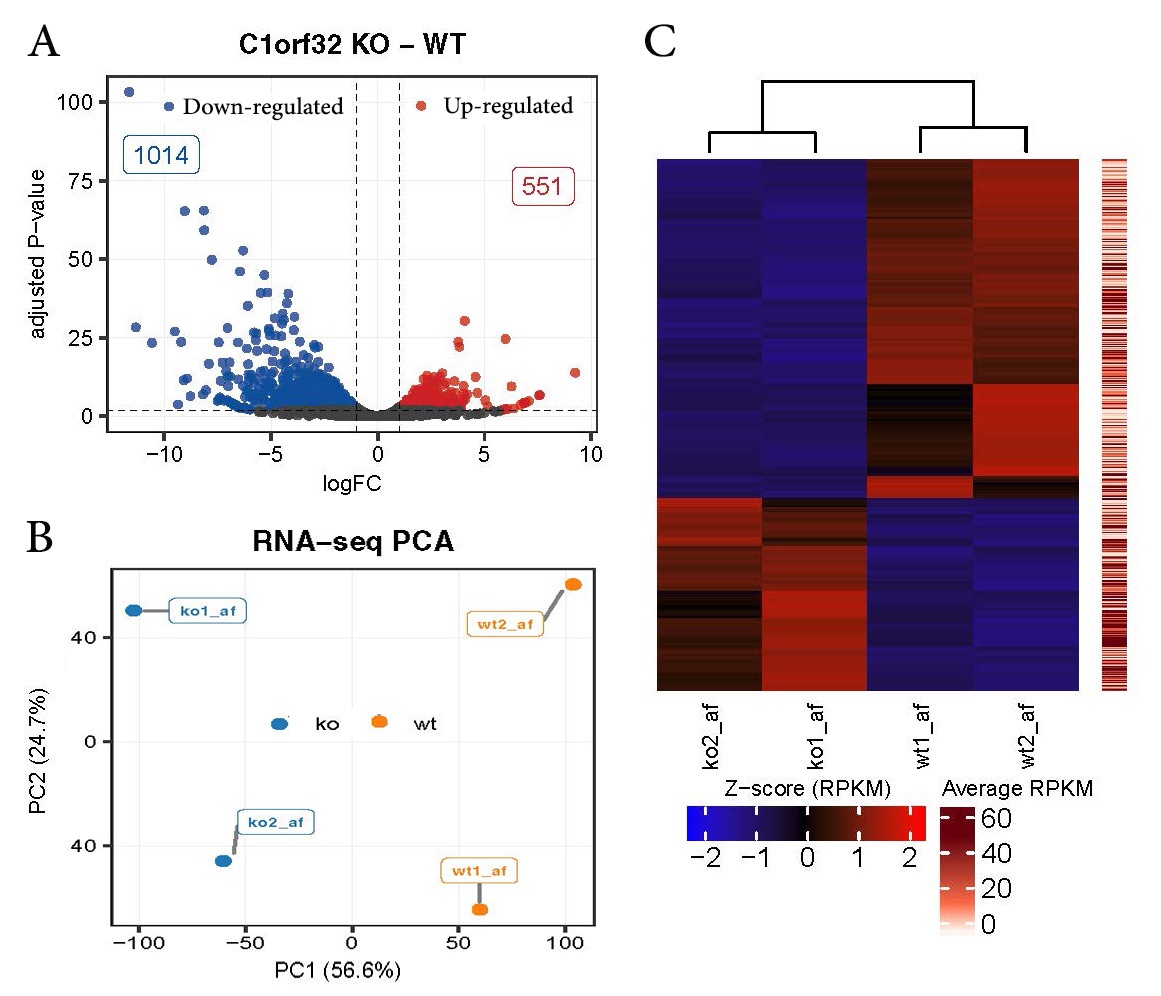

Supplement: Supplementary file 1 [file ijms-22-06768-s001.zip › ijms-1207723-SI.jpg]
